# Values of protected area landscapes shape the behaviors of subsistence users in Interior Alaska

**DOI:** 10.1007/s13280-025-02224-7

**Published:** 2025-08-09

**Authors:** Evan L. Salcido, Carena J. van Riper, William P. Stewart, Christopher M. Raymond, Henry S. Pollock

**Affiliations:** 1 Department of Natural Resources and Environmental Sciences, University of Illinois at Urbana-Champaign, S-524 Turner Hall, 1102 S. Goodwin Avenue, Urbana, IL 61801 USA; 2 School of Forest Resources, University of Maine, 223A Nutting Hall, Orono, ME 04469 USA; 3 Department of Recreation, Sport and Tourism, University of Illinois at Urbana-Champaign, 104 George Huff Hall, 1206 S. Fourth St., Champaign, IL 61820 USA; 4 Helsinki Institute of Sustainability Science (HELSUS), University of Helsinki, Viikinkaari 1, Biocentre 3, PO Box 65, 00790 Helsinki, Finland; 5 Department of Economics and Management, Faculty of Agriculture and Forestry, University of Helsinki, PO Box 65, 00790 Helsinki, Finland; 6 Ecosystems and Environment Research Program, Faculty of Biological and Environmental Sciences, University of Helsinki, PO Box 65, 00790 Helsinki, Finland; 7 Southern Plains Land Trust, P.O. Box 1016, Lamar, CO 81052 USA

**Keywords:** Behavior change, Environmental concern, Indigenous peoples and local communities, Protected areas, Social science

## Abstract

Diverse values expressed by people living around protected areas provide insight into shared reasons why agreements and tensions may exist yet are underrepresented in research with subsistence users. We administered a mixed-mode survey to residents living near Denali National Park and Preserve and Denali State Park in Interior Alaska, USA, to understand how values placed on landscapes by multiple interest groups could lead to actions benefitting the environment. We discovered four dominant groupings of specific values—centered on pristine nature, communal cohesion, relational fulfillment, and capacity growth—that signaled broad agreement on reasons why Denali landscapes are important. Results from a latent variable path model showed that these values worked in conjunction with previous experience to explain levels of environmental concern, and in turn, pro-environmental behavior. Communal cohesion, in particular, drove subsistence users’ concerns and behaviors, whereas pristine nature was the basis for behavioral decision-making among non-subsistence users.

**Supplementary Information:**

The online version contains supplementary material available at 10.1007/s13280-025-02224-7.

## Introduction

### Understanding the value basis of subsistence use

Integrating the values of local communities into protected area management is increasingly recognized as essential for generating more equitable and sustainable conservation outcomes. Benefits such as improved ownership of solutions produced and deeper commitment to sustained action (Frantzeskaki and Kabisch 2016) can be derived from both co-creation of knowledge in its multiple forms (Tengö et al. 2017) and broad expression of values that can reform policy and legal domains (Raymond et al. 2023). Environmental agencies and scholars have thus sought to understand how the values of multiple interest groups are ascribed to rapidly changing conditions within protected areas (Knapp et al. 2014; van Riper et al. 2019); however, examination of what it means to be ‘local,’ and the historical and social contexts in which individuals are situated in this corpus of knowledge, has been limited. Research concerning subsistence use—hunting or gathering wild, renewable resources for personal consumption (Udall 1980)—highlights a core difference in the lived experiences and historical treatment of interest groups, particularly Indigenous peoples that have lived off the land for multiple generations versus more recent arrivals who also engage in subsistence activities (Thompson 2008). Unearthing a more complete range of values expressed by all people who consume natural resources in different ways within the context of protected areas requires contextual knowledge to understand differences among self-ascribed identities (Tengö et al. 2017; Yletyinen et al. 2022), as well as empirical evidence of the inner pathways that connect values placed on landscapes to behavior change (Ives et al. 2020; Gould et al. 2023). Therefore, we sought to investigate how values placed on landscapes by subsistence users could lead to actions that benefit the environment.

Subsistence use practices have garnered attention from researchers and agencies alike, given the importance of defining and reflecting community members’ interactions with nature (e.g., Harmon 1987; Fall 1990). Although subsistence use is often framed as harvesting materials, there are multiple forms of subsistence practice that connect people, places and heritage (Gómez-Baggethun et al. 2013; Sahlins 2022). Subsistence use is an avenue for experiencing landscapes (Savo et al. 2016), for intergenerational and gendered bonding (Gartler 2018), for community-based food sovereignty (Anderson 2016) and an underlying reason for social conflicts concerning appropriate forms of human use (Jones et al. 2016; Raymond et al. 2023).

In the context of Alaska, subsistence use is practiced by both Alaska Natives (i.e., Indigenous peoples of the state) and non-Native people who have settled in the state more recently (referred to hereafter as “settlers”). Both groups practice intergenerational value transfers that embrace living off the land and stewarding wild resources, respecting all living things, and sharing resources with family and community, albeit to different degrees (Berkes 2009). Alaska Natives have been present for longer time periods and have consequently accrued different forms of landscape knowledge such as seasonal cycles of fish and wildlife (Twitchell 2005), while Alaskan settler outposts and communities relied on gathering food and raw materials from the landscape in the absence of conventional supply lines (Norris 2006). This historical dynamic resulted in state policies prioritizing subsistence use on federal lands and waters for Alaska’s rural population, a demographic encompassing settlers and Alaska Natives alike (Udall 1980; McGee 2010).

By Alaska state law and the Alaska National Interest Lands Conservation Act (ANILCA), subsistence is defined as customary and traditional uses of wild resources for various uses including food, shelter, fuel, clothing, tools, transportation, handicrafts, sharing, barter, and customary trade. However, many residents of Alaska self-identify as subsistence users irrespective of their legal recognition for traditional use by the US government (Department of the Interior 2024). Differences in state and federal laws,—i.e., whether any resident counts as a subsistence user, or only rural residents—reflect both Western and non-Western thinking at play in Alaska. For many Alaska Natives, subsistence is essential for cultural survival through food sovereignty, fortifying connections across households, and transferring knowledge and community vitality across generations (Gartler 2018). For settlers, meanwhile, subsistence may serve to supply individual households and friend groups with food, or as a social practice to reinforce a rural Alaskan identity. Environmental agencies in Alaska—particularly those within the Department of Interior—also function differently than in many international contexts, being actively involved in Tribal Consultation on decision-making activities both as a process and as mandated by ANILCA. The Alaskan context is therefore unique in its treatment for studying integration between environmental planning and broad representation of subsistence and non-subsistence users alike.

### Defining and measuring specific values

Specific values are garnering increased attention in the conservation sciences (Chan et al. 2018; Pascual et al. 2023; Raymond et al. 2023) and have longstanding theoretical traditions that span fields of study (Kenter et al. 2019). These types of values are grounded in specific places and contexts, ergo they can vary spatially and temporally across the landscape (Brown et al. 2020). Multiple meanings have been associated with landscape values, and sometimes their meanings overlap (Anderson 2016; Himes et al. 2023). Several studies that have looked at overlapping meanings associated with specific values in a participatory mapping context suggest more work is needed to unpack the concepts used to describe landscapes. For example, Biedenweg et al. (2019) questioned the viability of ‘recreation’ as a value, suggesting that other values such as ‘health’, ‘fun’, ‘heritage’ and ‘spirituality’ are masked by ‘recreation’. Likewise, Cerveny et al. (2017) delved into the assigned values used for special places and explored the qualitative data associated with assigned values used for special places, and found that a wide range of meanings were often associated with each value; subsistence, for instance, referred not only to traditional harvest activities (e.g., hunting, fishing) but overlapped with other values such as recreation, economic, and heritage.

Although often measured within individuals, specific values are rooted in the idea of transactionalism that assumes the processes of socialization and internalization influence how values take shape and are expressed by people (van Riper et al. 2018). Values as perceived and embodied by individuals are first shaped by communities, cultural groups, and societies, with cultural factors demonstrably affecting how individuals interpret and assign values to landscapes (Fagerholm and Käyhkö 2009). Specific values expressed by individuals may be aggregated by way of groups deliberating on their values to form a collective view, or combining specific values solicited from individuals to represent society (Raymond et al. 2014). Previous research has indicated that the prevalence of non-economic valuation that encompasses specific values is on the rise (Gross et al. 2023) and can effectively elicit a diverse range of values to understand behavioral decision-making (Johnson et al. 2023).

To measure specific values in ways that are relevant for environmental stewardship decision-making, previous research has developed typologies that span use and non-use objects of value (Rolston and Coufal 1991; Bengston 1994). In the participatory mapping scholarship, value typologies have been developed to elicit preferences for landscape conditions from community members and determine what matters most (Brown and Reed 2000). Though useful for answering applied research questions, this line of research has rarely evaluated the psychometric properties of specific values. One exception is Carr et al. (2022) who evaluated beliefs about landscape qualities as scaled survey items–rather than as a ranked typology–that could be evaluated for both reliability and validity. Researchers have also begun to disentangle and identify emergent themes within a specific value scale. Johnson et al. (2023), for instance, discovered that “nature-based” specific values encompassing wilderness, recreation, ecological integrity, and scientific qualities of places were most important for characterizing protected area landscapes in Alaska, and positively influenced behavioral intentions more than reported behavior. These nascent developments in the measurement of specific value scales need to be expanded to determine whether there are other dimensions that more completely reflect the richness and complexity of specific value concepts that can explain behavior change (Gould et al. 2023; Gross et al. 2023). New knowledge of the measurement properties of specific value scales would provide more robust and transferrable insights into how the transactional relationships between people and places are perceived by local communities.

### Behavior and its antecedents

Understanding and managing the behaviors of community members living around and within protected areas is complicated by the diverse array of actions that can be performed to express environmentalism. Pro-environmental behavior (PEB) encompasses not only private but also public and social spheres of goal-oriented actions that are intended to benefit the environment (Steg and Vlek 2009), each of which requires tailored intervention strategies (McKenzie-Mohr and Schultz 2014). Specifically, public-sphere behaviors encompass actions within the socio-political realm, such as petitioning, donating, or voting in favor of environmental causes (Schultz et al. 2005). Social sphere behaviors encompass interactive actions such as persuading, learning from, or educating others about environmental issues (Vaske and Kobrin 2001). Finally, private-sphere behaviors encompass actions taken at the individual or household level, such as recycling or avoiding personal travel to reduce carbon emissions (Andrade et al. 2022). With recent research showing that behavioral models are sensitive to the specific type of behavior being assessed (Larson et al. 2015; Milfont et al. 2018), a multidimensional conceptualization of PEB is increasingly recognized as essential to explain pertinent differences in how and why behavioral decisions are made. Research is thus needed to understand relationships among antecedents to PEB (Coon et al. 2020; Linder et al. 2022) and translate these relationships into actionable conservation outcomes which facilitate a more equitable and sustainable world (Pascual et al. 2023).

Previous research has indicated that the patterns of PEB can be explained by multiple psychological factors (Li et al. 2019; Ives et al. 2020). Environmental concern, defined as an attitudinal evaluation of facts, one’s own behavior, or the behavior of others that carry consequences for the environment (Fransson and Gärling 1999), is a primary driver of PEB (Nisbet et al. 2009). However, the optimal dimensions of environmental concern scales remain contested: Authors such as Schultz (2001) have argued for a tripartite conceptualization of concern organized around the self, other people, and the biosphere, while others (e.g., Best and Mayerl 2013) have posited there are cognitive (i.e., knowledge-based), affective (i.e., feeling-based), and conative (i.e., intention-based) dimensions of concern that reflect how people react to their environments and positively correlate with PEB. These concerns are rooted in human values (Schultz et al. 2005) but also experience use history (EUH) (Dietz et al. 1998; Gifford and Nilsson 2014). Specifically, EUH has been conceptualized in terms of two distinct but interrelated facets—level of exposure to an event and psychological interpretation of those events (Schreyer et al. 1984)—and measured with metrics such as total number of previous visits, total length of time visiting, or frequency of visitation to the area (Hammitt et al. 2004). While previous studies have recognized multiple dimensions of EUH (e.g., Budruk et al. 2008), other research has used fewer or even single metrics to effectively answer research questions in environmental contexts (Ibitayo and Virden 1996; Han et al. 2018). The use of EUH as a single item indicator therefore carries potential to explain the performance of PEB.

### Study purpose and hypotheses

**Fig. 1:**
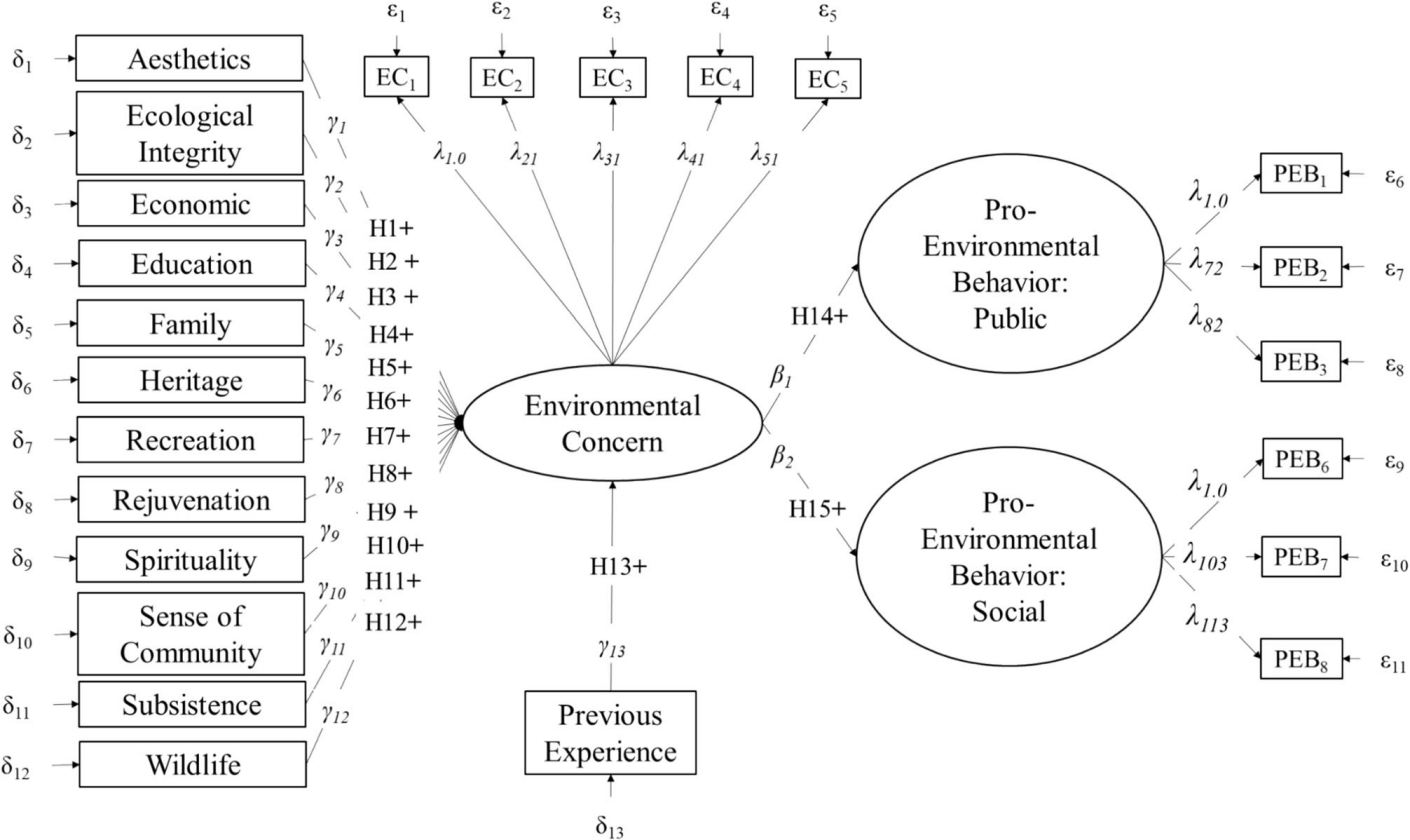
Hypothesized model of the theoretical relationships among twelve specific values, previous experience visiting Denali National Park and Preserve, environmental concern, and pro-environmental behavior. Squares represent observed variables, while circles represent latent variables. γ represent paths from observed to latent variables; λ represent paths from latent to observed variables; and β represent paths between latent variables. δ and ε represent measurement error for observed exogeneous and endogenous variables, respectively. H1–H15 represent fifteen hypothesized relationships between variables which were hypothesized to be positive (+)

We examined how involvement in subsistence use activities influenced the relationships among drivers of PEB among residents in Interior Alaska living near Denali National Park and Preserve and Denali State Park. The first phase of our study focused on understanding the behavioral antecedents of all survey respondents engaged in our research process. We examined the expression of specific values that reflected the reciprocal contributions from human-nature relationships, alongside previous experience and environmental concerns by testing fifteen hypotheses using our pooled sample. Specifically, we hypothesized that significant, positive relationships would be observed from specific values to environmental concern (H1—H12), from previous experience to environmental concern (H13), and from environmental concern to pro-environmental behavior (H14–H15; see Fig. 1). The relationships among these explanatory variables were then evaluated across subgroups of survey respondents that self-identified as subsistence or non-subsistence users. We tested the relationships among behavioral antecedents for residents defined by their resource consumption practices with the goal of understanding the meaning of subsistence use for all local residents.

## Materials and methods

### Researcher positionality

Our experiences and orientations as environmental social scientists shaped our research questions and process. Focusing on relationships between place-based values, concerns, and behaviors was spurred by the lead author’s personal interests and background as a human dimensions researcher. Given the lead author’s personal identification with a historically underrepresented group, promoting equitable environmental stewardship through our research was of particular concern. Our decision to examine how involvement in subsistence use activities influenced drivers of PEB was spurred by both interest in adding quantitative measurement to a topic frequently examined through a qualitative lens, and by previous qualitative research experiences in Interior Alaska which spurred recognition of the state’s novel subsistence use context compared to the contiguous USA (or “lower 48”). For instance, while our backgrounds predispose us toward framing the environment as a resource to be managed, many who identify as Alaska Natives would instead consider the environment a relative to be in relation to. We seek to advance environmental research by being cognizant of and adapting to these and other differences in positionality. We also acknowledge that, as researchers from a university founded through the Morrill Land-Grant Act of 1862, we have benefited from the theft of land and often violent removal of its Indigenous inhabitants.

### Study context

**Fig. 2:**
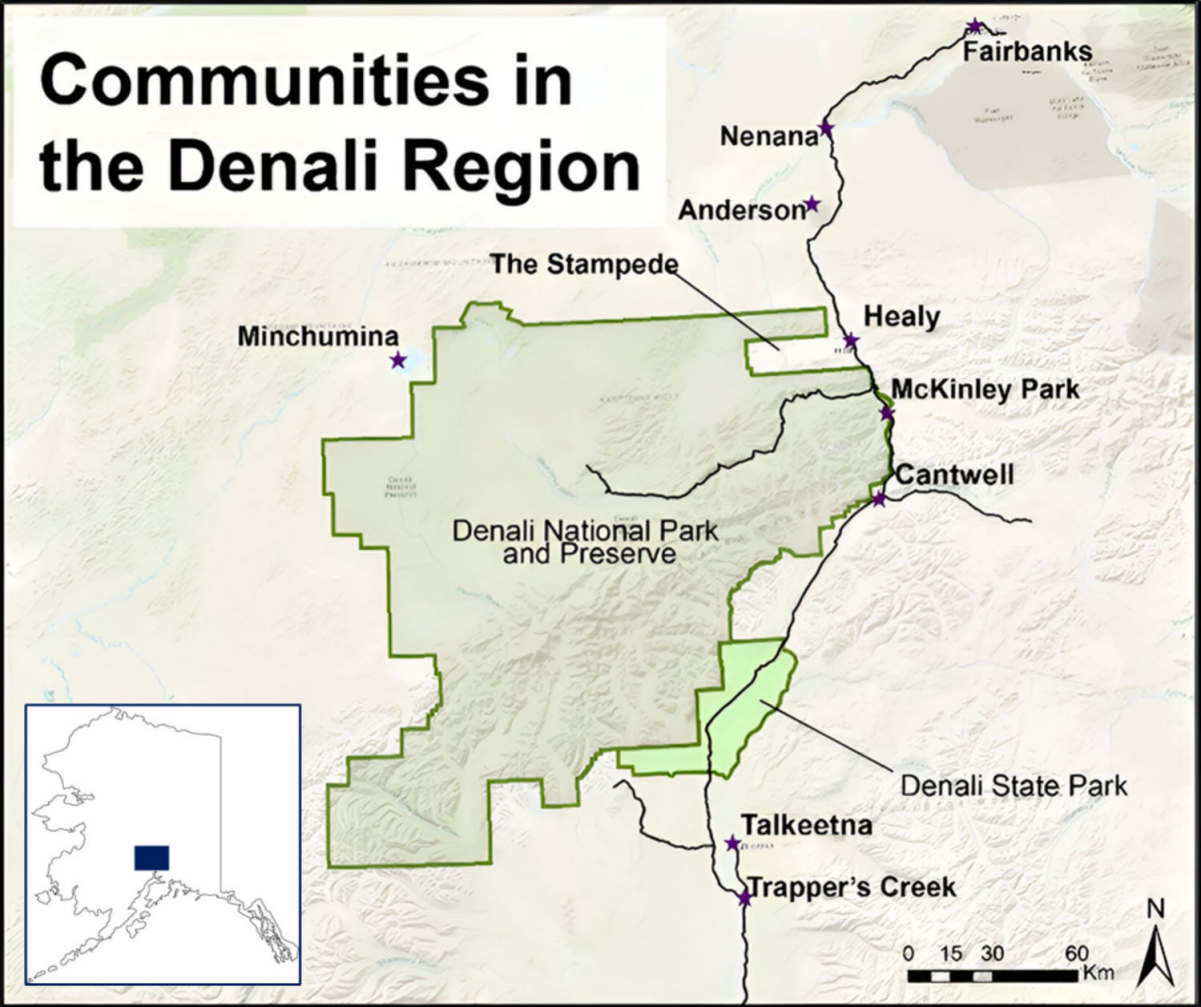
Map of the Denali study region, including the protected areas of Denali National Park and Preserve and Denali State Park and the surrounding communities (published with permission from Johnson et al. (2023))

The US state of Alaska provided an ideal setting to study variation in subsistence use activities given the importance of resource consumption for local and state-based economies, as well as the cultures and practices of many people throughout the state (Wolfe 2004). Our research took place in the region surrounding Denali National Park and Denali State Park in Interior Alaska (see Fig. 2): Containing over six million acres of land and the highest mountain peak in North America, these protected areas are crucial sources of tourism-based livelihood for many nearby communities (Denali Borough 2019). Hereafter referred to as the Denali region, this landscape hosts approximately 1600 permanent residents across an extensive network of subarctic ecosystems that have undergone little industrial development but remain vulnerable to anthropogenic activity (Savo et al. 2016). The Denali region also intersects the traditional lands of five different groups of Alaska Natives—the Ahtna, Dena’ina, Koyukon, Upper Kuskokwim, and Tanana peoples—who are part of the larger Athabascan language family (Haynes and Simeone 2021); just over seven percent of Denali region residents identify as Alaska Native (US Census Bureau 2025).

We engaged residents from 10 communities located along the George Parks Highway, which connects the cities of Anchorage and Fairbanks and provides a primary transportation corridor through the Denali region. Our process was guided by previous research (e.g., Knapp et al. 2014) and an Executive Committee that included 10 members of regional communities and local organizations such as the Denali Borough, National Park Service, Holland America/Princess, and the Alaska Department of Natural Resources. Local rural residents of the Denali region—particularly within four subsistence resident zone communities of Cantwell, Lake Minchumina, Nikolai, and Telida—who have customarily and traditionally used what are now park lands for subsistence purposes are authorized to practice activities such as hunting, fishing, trapping, and timber gathering within Denali National Park (Haynes and Simeone 2021). Three of these communities—Lake Minchumina, Nikolai and Telida—are off Alaska’s road system and therefore do not have direct access to transportation corridors and their associated resources and employment opportunities. Importantly, residents from the predominantly Native communities of Nikolai or Telida were not engaged in our survey research process due to limited accessibility, which carries important implications for interpretation of our study findings and recommendations for policy.

### Data collection and survey measures

Our survey data collection process was preceded by one year of qualitative research including a stakeholder analysis and interviews (Salcido et al. 2023), as well as focus groups  (Johnson et al. 2023). These steps were taken to better understand the study context, cultivate local partnerships, and develop appropriate methodology. We followed up on this relationship-building process by administering a mixed-mode survey to 95% of P.O. boxes and household addresses situated along or near the George Parks Highway between Nenana and Talkeetna, residents of Lake Minchumina**,** and a smaller proportion of residents living in one zip code west of Fairbanks that comprised 9.7% of the total sample. Our sampling frame was purchased from the US Postal Service and included 3000 residents who were registered across eight zip codes in the study region. Following standard procedures from Dillman et al. (2014) to maximize response rates, our mailback survey was administered in three waves. During initial contact, residents received a packet containing the questionnaire and an introductory letter. Recipients were invited to complete and return the questionnaire by mail using a postage paid envelope or to use an ID number that would allow them to participate in an online survey presented using the Qualtrics software platform. After one week, all residents who did not respond to the initial mailing were sent a reminder postcard. After two weeks, packets containing another copy of the questionnaire and a final cover letter were distributed to all non-respondents.

Multiple steps were taken to encourage participation in our study. First, we developed our research questions to address relevant local concerns and emphasized how contributing one’s own perceptions and voice would shape final reports that we would share with agency decision-makers. Second, our research materials were personalized with hand-addressed envelopes and cover letters personally signed using blue ink (McCoy and Hargie 2007). Third, the questionnaire and introductory letters were printed in color and presented as professionally formatted booklets. Fourth, we used distinctly colored envelopes (i.e., brown, purple, and green) to increase the likelihood that potential respondents would notice our request in the mail (Dillman et al. 2014). Fifth, we raised awareness of the survey by posting flyers in public venues (e.g., post offices, a library, community centers), announcing the survey in meetings, a newsletter, on the radio, and on social media. Finally, members of our Executive Committee encouraged participation from people at their places of employment and we personally contacted all 65 people who had participated in earlier qualitative phases of our study to ask them to consider participating in the mailback survey.

The questionnaire developed for this study contained multiple batteries of questions informed by previous research and tailored to the study context (see Appendix 10.1007/s13280-025-02224-7). We measured private (i.e., individual), public (i.e., socio-political), and social (i.e., interactive) dimensions of PEB by asking survey respondents to rate how frequently they engaged in specific types of activities (van Riper et al. 2019). Environmental concern was measured using multiple questions about cognition (i.e., thoughts), affect (i.e., feelings), and conation (i.e., convictions) that collectively comprised a single latent variable (Cruz and Manata 2020). Specific values were measured by asking survey respondents to agree or disagree with statements that reflected reasons why places in the Denali region could be considered special. Our specific value statements were derived from the following landscape qualities established by Brown and Reed (2000) and adapted to the study context (van Riper et al. 2020): economic, subsistence, education, recreation, family, rejuvenation, sense of community, heritage, spirituality, aesthetics, ecological integrity, and wildlife. Finally, we used previous visits to Denali National Park and Preserve as a proxy for measuring experience use history, by asking respondents to self-report the number of their previous visits to Denali National Park and Preserve in their lifetime (Han et al. 2018). To evaluate subsistence use, we provided a written definition of subsistence use—hunting or gathering wild, renewable resources for personal consumption—followed by asking whether a respondent self-identified as a subsistence user. This study was reviewed and approved by the University of Illinois Institutional Review Board (protocol number 18679), including permission to engage with vulnerable populations and formal documentation indicating support for our research to be conducted with the communities of interest.

### Data analysis

All survey data were entered, cleaned, and analyzed using descriptive statistics. Data were evaluated for missingness (see Appendix 10.1007/s13280-025-02224-7) using Little’s (1988) global test—according to guidelines provided by Enders (2022)—and were found to be not missing completely at random (MCAR) (*p* < 0.05). The missing data patterns were designated missing at random (MAR), so full-information maximum likelihood (FIML) was used. Data were further evaluated for normality, skewness, and kurtosis (see Appendix 10.1007/s13280-025-02224-7), with the Shapiro–Wilk test (Shapiro and Wilk 1965) indicating evidence of significant non-normality for specific value, park visitation, environmental concern, and PEB variables (all *p* > 0.01). The robust maximum likelihood (MLR) method was therefore applied to statistically correct standard errors (Satorra and Bentler 2001).

We tested for collinearity among the 12 specific values (see Appendix 10.1007/s13280-025-02224-7) and used a principal component analysis (PCA) to identify any emergent dimensions within the data and increase interpretability (Manly and Navarro Alberto 2016). We employed Kaiser-Varimax rotation and retained components from the PCA with an eigenvalue greater than 1.0 (Osborne 2015). Components were interpreted based on their variable loading scores, using a factor loading cutoff value of 0.4 (Hair et al. 2010) under the assumption that variables generating the largest factor loadings for each component would be most influential in defining its characteristics.

After determining the appropriate configuration of specific value items, a two-step modeling approach was adopted, with confirmatory factor analysis (CFA) performed to test validity and reliability of measurement items, followed by structural regression modeling to test whether the data fit the study hypotheses (Anderson and Gerbing 1988). Direct path coefficients from predictors to dependent variables were evaluated to understand the structural relationships among the latent variables. Model fit was assessed using Comparative Fit Index (CFI) and Standardized Root Mean Square Residual (SRMR), with CFI < 0.90, and SRMR < 0.08 that were considered acceptable (Kline 2023). Models were estimated and fit was assessed separately for the pooled sample and then for subsistence and non-subsistence user subgroups. Measurement invariance across the two subgroups was established through an invariance constraints procedure and a chi-square difference test (Putnick and Bornstein 2016). All data were analyzed using R 4.2.2 and SPSS Statistics 28.0. Basic.

## Results

### Survey sample and socio-demographic characteristics

The final sample consisted of 313 survey respondents after removing duplicates and invalid addresses (i.e., “return to senders” due to vacancies or inability to deliver mail to resident), yielding a response rate of 12.1% (see Table 1). Respondents were closely split between male (53.80%) and female (46.20%), and on average were 55.20 years old (*SD* = 15.09 years). Most (72.42%) respondents reported having obtained a two-year college degree or higher, and most households (76.47%) earned less than $100 000 per year. Racially, most respondents identified as White (84.98%), with 7.99% identifying as American Indian or Alaska Native. More than half (n = 215, 68.69%) of survey respondents identified as subsistence users. No significant differences were observed between subsistence and non-subsistence users concerning gender (χ^2^ [305, N = 301] = 1.04, *p* = 0.31), age (t[181.18] = 0.99, *p* = 0.33), education (χ^2^ [5, N = 301] = 0.76, *p* = 0.98), income (χ^2^ [4, N = 272] = 8.00, *p* = 0.09), or previous experience (t[123.49] = 0.45,* p* = 0.65).

**Table 1:** Socio-demographic characteristics and previous experience visiting Denali National Park and Preserve for the pooled sample of survey respondents, subsistence users, and non-subsistence users. ^1^Respondents could check all that applied so column totals may not equal 100%. ^2^Data points which exceeded UQ + (1.5*IQR) were removed prior to calculating M and SD

Variable	Pooled sample (n = 313)	Subsistence users (n = 215)	Non-subsistence users (n = 98)
*Gender (%)*			
Male	53.80	55.98	48.96
Female	46.20	44.02	51.04
Age [years; M (SD)]	55.20 (15.09)	54.60 (15.00)	56.47 (15.30)
*Education (%)*			
Less than high school	0.33	0.48	0.00
High school graduate	16.94	16.27	18.48
Vocational/trade school certificate	10.30	10.53	9.78
Two-year college degree	39.53	40.19	38.04
Four-year college degree	6.31	6.22	6.52
Graduate degree	26.58	26.32	27.17
*Income (%)*			
Less than $49 999	42.28	46.24	33.72
$50 000 to $99 999	34.19	33.33	36.05
$100 000 to $149 999	15.07	13.98	17.44
$150 000 to $199 999	5.15	4.84	5.81
Greater than $200 000	3.31	1.61	6.98
*Race*^*1*^* (%)*			
American Indian or Alaska Native	7.99	8.37	7.14
Asian	2.56	2.33	3.06
Black or African American	2.87	3.72	1.02
Native Hawaiian or other Pacific Islander	1.28	1.40	1.02
White	84.98	82.79	87.76
Other	7.35	7.44	7.14
Previous Experience^2^ [M (SD)]	28.41 (37.73)	27.59 (35.29)	30.15 (42.65)

Comparing our survey socio-demographics with US Census data indicates a broadly representative sample of the population of Denali Borough. As of July 2023, Denali Borough had a population of 1584 (45.00% female), with a median age of 44.1 years and median household income of $88 935 per year. Racially, most residents of Denali Borough identify as White (76.3%), with 7.3% identifying as American Indian or Alaska Native (US Census Bureau 2025).

### Specific values

The pooled sample of respondents most strongly agreed that the specific values of Wildlife (*M* = 4.61, *SD* = 0.71) and Recreation (*M* = 4.59, *SD* = 0.60) were associated with places in the study region. Conversely, respondents least strongly agreed that places embodied Economic qualities (*M* = 3.33, *SD* = 1.10) and Spirituality (*M* = 3.48, *SD* = 1.13). When comparing between subgroups, subsistence users reported significantly stronger agreement with survey items that measured Subsistence (t[146] = 8.43, *p* < 0.05) and Rejuvenation (t[146] = 2.65, *p* < 0.05) values than non-subsistence users (see Table 2).

**Table 2:** Means and standard deviations (SDs in parentheses) of specific values items according to survey respondents in the pooled sample, subsistence users, and non-subsistence users. *Note*. All items were measured on a Likert scale where 1 = “Strongly Disagree” and 5 = “Strongly Agree.” *p < 0.05 for comparison between subsistence users and non-subsistence users

Specific values	Pooled sample (n = 313)	Subsistence users (n = 215)	Non-subsistence users (n = 98)
*Pristine Nature*	4.51 (0.63)	4.54 (0.62)	4.45 (0.65)
Aesthetics: A place that has attractive scenery, sights, sounds, or smells that cannot be experienced anywhere else	4.54 (0.77)	4.56 (0.77)	4.49 (0.79)
Ecological Integrity: A place that has intact ecosystems with the ability to support and maintain ecological processes	4.37 (0.80)	4.39 (0.82)	4.33 (0.74)
Wildlife: A place inhabited by wildlife unique to Alaska	4.61 (0.71)	4.63 (0.69)	4.54 (0.76)
*Communal Cohesion*	3.81 (0.70)	3.91 (0.71)*	3.60 (0.66)*
Heritage: A place with history and traditions that are passed down to future generations	3.65 (1.01)	3.70 (1.04)	3.54 (0.92)
Spirituality: A place that is sacred, religious, or spiritually significant	3.51 (1.12)	3.54 (1.14)	3.45 (1.10)
Sense of community: A place where I have close relationships with other members of my community	3.97 (1.01)	4.01 (1.01)	3.88 (1.01)
Subsistence: A place to harvest food or other resources to sustain my life and that of my family	4.10 (0.86)	4.37 (0.71)*	3.50 (0.87)*
*Relational Fulfillment*	4.45 (0.57)	4.52 (0.53)*	4.31 (0.64)*
Family: A place where I can spend time with my family	4.25 (0.89)	4.31 (0.86)	4.11 (0.96)
Recreation: A place where I can pursue recreation activities	4.59 (0.60)	4.63 (0.55)	4.49 (0.68)
Rejuvenation: A place where I can feel better physically and/or mentally	4.53 (0.76)	4.61 (0.69)*	4.34 (0.87)*
*Capacity Growth*	3.67 (0.77)	3.65 (0.79)	3.71 (0.75)
Economic: A place to earn income for employment	3.36 (1.10)	3.31 (1.09)	3.45 (1.10)
Education: A place to learn about, teach, or research the environment and people	3.98 (0.88)	3.99 (0.89)	3.96 (0.87)

**Fig. 3:**
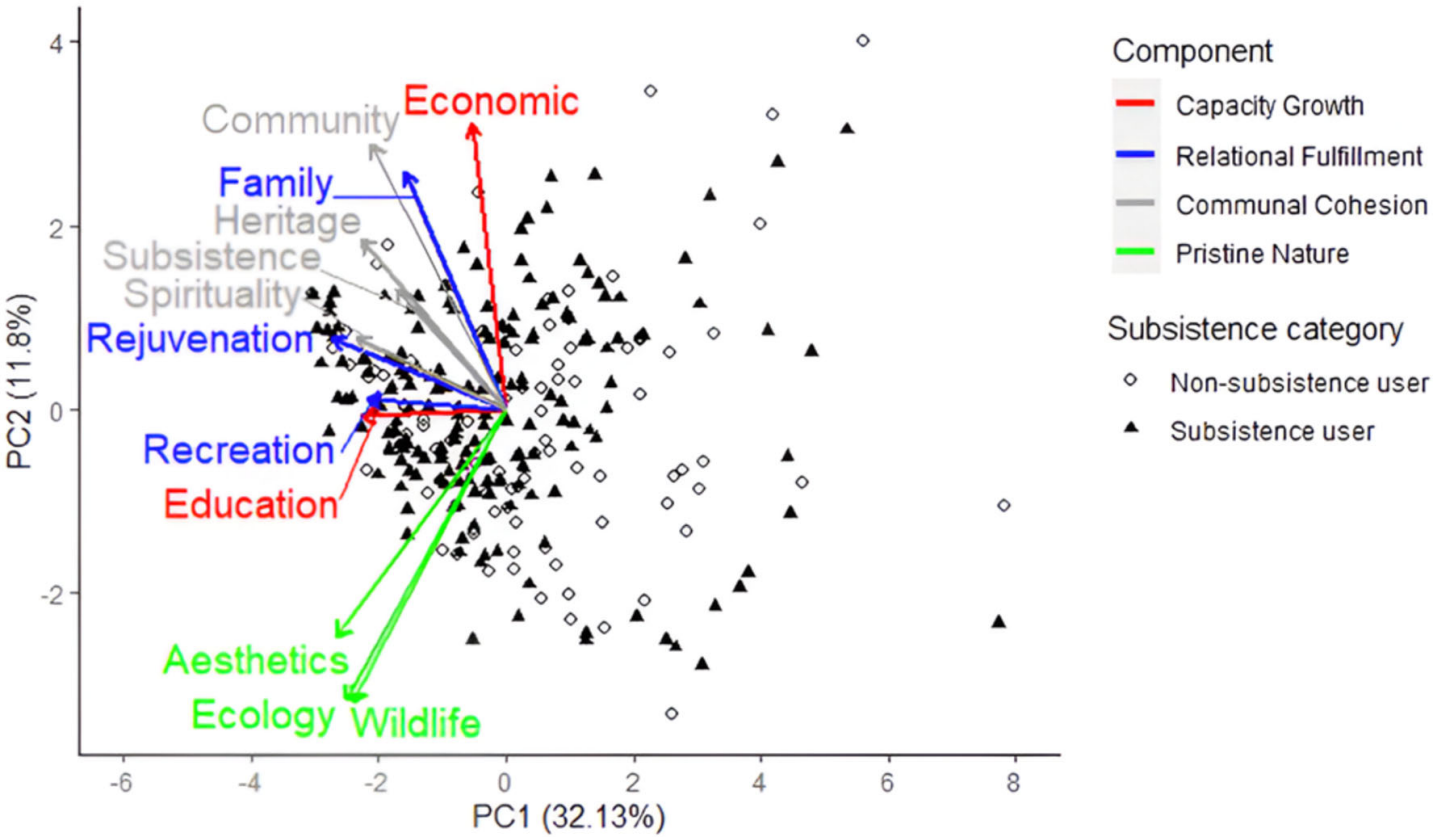
Results from a principal component analysis biplot for the specific values data, demonstrating clustering among twelve individual items into four groupings of specific values. Points on the biplot represent individual observations among survey respondents, while vectors represent direction and impact of specific values on the principal components

Results from the PCA enabled us to reduce the 12 specific values into four groupings accounting for 62% of variance. Based on clustering of specific values (see Fig. 3), these four groupings were labeled 1) Pristine Nature; 2) Communal Cohesion; 3) Relational Fulfillment; and 4) Capacity Growth (see Appendix 10.1007/s13280-025-02224-7). Individuals with high Pristine Nature scores placed high value on the ecological integrity, unique wildlife species, and natural aesthetics of Alaska. A high Communal Cohesion score indicated valuing generational heritage, spiritual practices, sense of community, and life-sustaining subsistence practices within Alaska. A high Relational Fulfillment score indicated valuing recreational activities, time spent with family, and being physically or mentally refreshed by Alaskan landscapes. Finally, individuals with high Capacity Growth scores valued earning income and learning or teaching others about Alaska. On average, Communal Cohesion was significantly higher for subsistence (*M* = 3.91, *SD* = 0.71) than non-subsistence users (*M* = 3.60, *SD* = 0.66), and Relational Fulfillment was also higher for subsistence (*M* = 4.52, *SD* = 0.53) versus non-subsistence users (*M* = 4.31, *SD* = 0.64).

Three instances of cross-loading were observed during the PCA. The specific value of Subsistence was most strongly associated with Communal Cohesion but had a factor loading score of 0.40 on Relational Fulfillment. Education had its highest loading on Capacity Growth with cross-loading of 0.50 on Pristine Nature. Sense of Community had its highest loading on Communal Cohesion with a cross-loading of 0.45 on Relational Fulfillment. Subsistence and Sense of Community were retained within the final model based on connections between subsistence practices and sense of community which are supported by existing literature (e.g., Wolfe 2004; Berkes 2009). Education was also retained given that it works in conjunction with economic values—the other specific value included in this grouping—to support Capacity Growth of local communities.

### Confirmatory factor analysis results

Fit indices from our CFA indicated acceptable model fit (χ^2^ = 130.064, df = 41; CFI = 0.954; SRMR = 0.064). All factor loadings were ≥ 0.40 (see Table 3). Reliability and validity were examined, and low internal consistency (α = 0.56) was observed among items measuring private-sphere PEB. Subsequent exploratory factor analysis of PEB items was therefore conducted using maximum likelihood extraction and Kaiser-Varimax rotation methods. Results showed that a two-factor conceptualization of PEB would account for 46% of the total variance. Consequently, the private-sphere behavior dimension and associated items were dropped from the model. Reliability for the remaining scales was acceptable, with alpha ranging from 0.74 to 0.92 (Hair et al. 2010) and composite reliability ranging from 0.72 to 0.92 (Bagozzi and Yi 1988).

**Table 3:** Means (SDs in parentheses), factor loading scores (λ), Cronbach’s alpha (α) coefficients, and composite reliability (Ω) for survey items measuring environmental concern and pro-environmental behavior for the pooled sample of survey respondents, subsistence users, and non-subsistence users. ^1^Measured on a Likert scale where 1 = “Strongly Disagree” and 5 = “Strongly Agree.” ^2^Measured on a Likert scale where 1 = “Very Rarely” and 5 = “Very Frequently.”

Variable	λ	Pooled sample	Subsistence users	Non-subsistence users
*Environmental concern*^*1*^* (α = 0.92, Ω = 0.92)*				
EC_1_: It bothers me when I think about the environmental conditions in which our children and grandchildren will probably have to live	0.81	4.08 (1.00)	4.09(1.03)	4.06 (0.92)
EC_2_: If we continue down the same path, we are heading toward an environmental catastrophe	0.92	3.93 (1.19)	3.91 (1.23)	3.97 (1.10)
EC_3_: Decision-makers are doing far too little to protect the environment	0.91	3.98 (1.18)	3.96 (1.22)	4.03 (1.07)
EC_4_: To protect the environment, we should all be willing to reduce our current standard of living	0.82	3.48 (1.32)	3.46 (1.35)	3.52 (1.27)
EC_5_: There are limits on growth that our industrialized world has already exceeded or will soon reach	0.70	3.82 (1.06)	3.81 (1.08)	3.84 (1.02)
*Pro-Environmental Behavior: Public*^*2*^* (α = 0.79, Ω = 0.83)*				
PEB_1_: Participated in a policy process like a public comment period that affected the environment	0.74	2.51 (1.20)	2.62 (1.21)	2.28 (1.17)
PEB_2_: Donated money with the intention of benefiting the environment	0.64	2.49 (1.22)	2.54 (1.21)	2.38 (1.24)
PEB_3_: Wrote a letter or email about an environmental issue	0.91	2.21 (1.24)	2.29 (1.26)	2.02 (1.16)
*Pro-Environmental Behavior: Social*^*2*^* (α = 0.74, Ω = 0.72)*				
PEB_6_: Encouraged other people to attend an event related to the environment	0.85	2.23 (1.12)	2.28 (1.10)	2.11 (1.16)
PEB_7_: Talked to other people about the environment	0.64	3.72 (1.09)	3.73 (1.11)	3.68 (1.03)
PEB_8_: Learned from other people like longtime residents or Elders to solve an environmental problem	0.56	2.73 (1.12)	2.80 (1.11)	2.57 (1.14)

### Model comparison

**Fig. 4:**
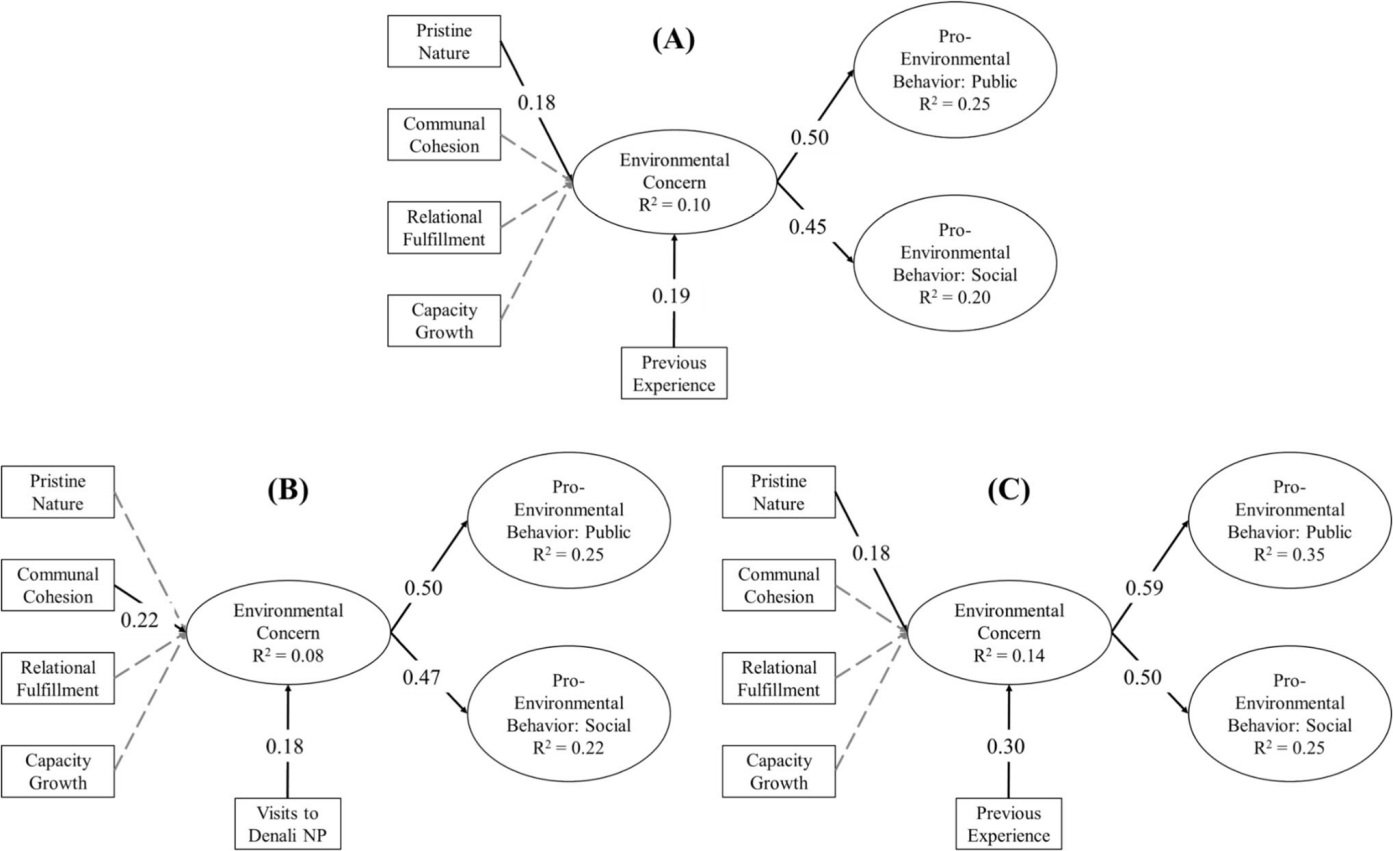
Results from the structural regression model of relationships among specific values groupings, previous experience visiting Denali National Park and Preserve, environmental concern, and pro-environmental behavior for **A** the pooled sample, **B** subsistence users (n = 215) [model fit: χ2 = 152.369, df = 61; CFI = 0.931; SRMR = 0.077], and **C** non-subsistence users (n = 98)[model fit: χ2 = 105.012, df = 77; CFI = 0.919; SRMR = 0.083]. Significant (*p* < 0.05) hypothesized paths and coefficients are indicated by solid black lines. Non-significant hypothesized paths are shown as gray dashed lines

Structural regression modeling revealed partial support for the hypothesized relationships in our path model (see Fig. 4) and indicated acceptable fit of the model to the data (χ^2^ = 232.888, df = 91; CFI = 0.919; SRMR = 0.075). In the pooled sample, Pristine Nature (β = 0.18) and Previous Experience (β = 0.19; H13) significantly and positively predicted Environmental Concern. In turn, Environmental Concern significantly and positively predicted PEB in both the public (β = 0.50; H14) and social spheres (β = 0.45; H15).

We compared subgroups defined by their self-identification as a subsistence user or non-subsistence user. Specifically, factor means were compared across both subgroups (see Table 4); subsistence users had significantly higher scores for Communal Cohesion (*M* = 3.91, *SD* = 0.71) and Relational Fulfillment (*M* = 4.52, *SD* = 0.53), as well as public-sphere pro-environmental behavior (*M* = 2.49, *SD* = 1.04) compared to non-subsistence users (*M* = 3.60, *SD* = 0.66; *M* = 4.31, *SD* = 0.64; and *M* = 2.23, *SD* = 0.97). Measurement invariance was established by the invariance constraints procedure and chi-square difference testing (Δχ^2^ = 12.77, Δdf = 11, p = 0.64; see Appendix 10.1007/s13280-025-02224-7). Finally, regression coefficients were compared among both subgroups; significant positive relationships were observed among Previous Experience, Environmental Concern, and both spheres of PEB for subsistence and non-subsistence users. Relationships between the four specific values and Environmental Concern varied. For subsistence users, Communal Cohesion was the only significant predictor of Environmental Concern (β = 0.22, *p* < 0.05) whereas Pristine Nature was the only significant predictor of Environmental Concern for non-subsistence users (β = 0.18, *p* < 0.05).

**Table 4:** Structural regression modeling results and Analysis of Variance (ANOVA) results for each construct that was compared across the pooled sample, subsistence users, and non-subsistence users. *Note.* Eta squared (η2) provides a measure of effect size (i.e., the ratio of variance explained by the independent variable) and ranges from 0 to 1. * Subsistence users and non-subsistence users significantly different at *p* < 0.05

Variable	Pooled sample	Subsistence users	Non-subsistence users	*F*-value	*P*-value	η^2^
Pro-environmental behavior: public	2.41 (1.03)	2.49 (1.04)	2.23 (0.97)	4.201	0.041*	0.014
Pro-environmental behavior: social	2.89 (0.90)	2.94 (0.89)	2.78 (0.90)	2.149	0.144	0.007
Environmental concern	3.88 (0.99)	3.85 (1.02)	3.88 (0.93)	0.029	0.865	0.000
Previous experience	28.41 (37.73)	27.59 (35.29)	30.15 (42.65)	0.319	0.573	0.001
Pristine nature	4.51 (0.63)	4.54 (0.62)	4.45 (0.65)	1.170	0.280	0.004
Communal cohesion	3.81 (0.70)	3.91 (0.71)	3.60 (0.66)	11.944	0.001*	0.040
Relational fulfillment	4.45 (0.57)	4.52 (0.53)	4.31 (0.64)	8.677	0.003*	0.028
Capacity growth	3.67 (0.77)	3.65 (0.79)	3.71 (0.75)	0.389	0.533	0.001

## Discussion

### Research implications

This study revealed how an array of specific values gave rise to pro-environmental behaviors in the Denali region of Alaska and is the first to provide empirical evidence of the value differences among local residents that define themselves in accordance with their resource consumption activities. We found that the specific values of subsistence and non-subsistence users differed and worked in conjunction with previous experience to explain environmental concern, and in turn, PEB. Concerns about the negative impacts of environmental degradation on communal cohesion drove PEB of subsistence users, whereas non-subsistence users were more likely to engage in PEB due to environmental degradation per se and the loss of pristine nature. These results were generated to better understand what it means to be a local resident who practices subsistence in the Denali region and conduct a more plural, non-economic valuation by showcasing the distinguishable value basis of residents surrounding protected areas (Gross et al. 2023). We also advance knowledge of how specific values that are unique to different interest groups can catalyze changes in environmental concern, and in turn, behavioral intentions to benefit public and social spheres.

It is our hope that the value pathways identified in this study can be more fully recognized and activated through interventions such as deliberation and negotiation across interest groups. The four dominant groupings of values that we discovered—pristine nature, communal cohesion, relational fulfillment, and capacity growth—suggest relatively broad social agreement on these topics in accordance with previous qualitative research (e.g., Salcido et al. 2023). For example, parallels can be drawn between values of pristine nature, communal cohesion, and relational fulfillment, and place meanings such as landscape of wildlife habitat (e.g., “*you can’t know what Alaska is unless you get out…[where] there’s animals*”), distinct sense of community (e.g., “*The reason I moved up here, for the strong sense of community, that is still here. And therefore, I am still here*.”), and wildland areas tied to recreation (e.g., “*there’s not a lot of people [in the lower 48] who have access to wilderness like that*.”). These shared values could thus become the basis for participatory processes (e.g., facilitated community discussions) pursued by resource management agencies to foster social exchange, learning, and trust building in a personal and bi-directional fashion (Raymond et al. 2022; Andrade et al. 2023) to unite diverse communities around protected areas.

The value bases for PEB that we discovered gave rise to environmental concerns of subsistence versus non-subsistence users. That is, two subsets of respondents who participated in our study reported similar demographics but differed in their value positions and self-identification as subsistence users. Our finding that Communal Cohesion positively influenced environmental concern among subsistence users—but not among non-subsistence users—supports and extends previous research suggesting that positionality and access to resources influence collective valuation of landscapes, and that connections to places and heritage values are ingrained in resource consumption activities (e.g., Fagerholm and Käyhkö 2009; Gómez-Baggethun et al. 2013). Indeed, the relationships that form between subsistence users and places give rise to and are reinforced by reciprocal contributions between people and nature (Ojeda et al. 2022). This finding also underscores the importance of the Alaskan identity that can be rooted in ideals of self-reliance and frontier individualism (Hébert 2014), and that subsistence users may be seeking solitude—a key characteristic of communal cohesion. We posit that subsistence is a way of life for most Alaskans, coupled with a sense of bonding with others in a similar context, to partially explain the influence of communal cohesion and increased levels of intended public-sphere PEB among residents that subsist off the land. Additionally, and in line with previous research involving visitors to Denali National Park and Preserve (Johnson et al. 2023), our pooled sample of all survey respondents showed that the aesthetic quality of places and integrity of ecosystems and wildlife populations reflected why Interior Alaska could be considered special. Such specific values were universally important among residents who participated in this study and visitors.

Nevertheless, prioritization by subsistence users of communal cohesion over pristine nature recapitulates important but often subtle differences between subsistence values and wilderness values, particularly in the context of protected areas in Alaska. For example, Dear and Myers (2005) found that recreationists interviewed at Gates of the Arctic National Park had conflicting and often contradictory opinions about subsistence vs. wilderness values, and were unable to integrate the two ideas into a coherent conceptual structure. A review of subsistence management in Alaska National Parks identified conflicts involving wilderness values and subsistence values between National Park Service staff and Indigenous Alaskan communities, with Indigenous respondents highlighting increased self-determination and sovereignty over subsistence practices as their primary issue (Green et al. 2022). Ultimately, climate change could exacerbate these conflicts given its potential negative impacts on both pristine wilderness and wildlife populations that subsistence users depend on, particularly in places like the Arctic where the absolute effects of climate change are projected to be greatest (Dunn 2024). Considering the specific needs of subsistence users will be useful for agencies tasked with engaging such diverse audiences that have different histories and relationships with the government.

We responded to calls from previous research to advance the theoretical associations among the social psychological drivers of PEB (Dietz et al. 1998; Gifford and Nilsson 2014), particularly in the context of protected areas (van Riper et al. 2019). Our observations that previous visits to a protected area significantly and positively predicted environmental concern and PEB support increased experience with specific sites begetting bonding to these sites and sensitivity to deteriorating conditions (supporting White et al. 2008). This result stands to reason because the state and federally designated protected areas in the Denali region are deeply integrated into the everyday life of local communities, with residents frequently interacting with the protected area in deeply personal and meaningful ways (Salcido et al. 2023). Relatedly, previous experience was nearly twice as important for explaining levels of environmental concern among non-subsistence users. This may be a consequence of subsistence use—and associated knowledge and skill levels—facilitating experience with natural areas beyond the bounds of Denali National Park and Preserve. That is, experiencing a protected area could represent a smaller part of subsistence users’ overall experiences in the outdoors compared to non-subsistence users, who may be more likely to experience nature within park boundaries. Our results also indicate that environmental concern across all interest groups can be fostered among residents as an effective means of promoting behavior change (Linder et al. 2022). For example, resource managers could build environmental concern by not only investing in inclusive participatory processes that build knowledge and enhance connections with local landscapes (Goodson et al. 2022), but also communicating in ways that: (a) encourage individuals to ascribe responsibility to themselves; (b) build confidence in their ability to make change, and (c) identify ways that environments can be changed as a result of individual behavior (Heberlein 2012).

Specific values provided a conceptual basis for understanding environmental concerns and subsequent actions that minimized degradation to natural areas. We observed that residents’ values were not exclusively based on commodities or livelihood per se but rather on maintaining the integrity and natural beauty of Alaska’s ecosystems, while fostering communal cohesion, relational fulfillment, and capacity for growth and change. In line with a growing body of research focused on the role of specific values in explaining behavior in environmental management contexts (e.g., van Riper et al. 2019; Gould et al. 2023; Johnson et al. 2023), we argue that shared meanings of places influence the actions people take to protect those places, which, in turn, may foster stewardship and attachments formed between people and places (Thomas et al. 2024; Garcia-Martin et al. 2018). These interrelated concepts thus carry potential to inform future place-based research that aims to represent a range of interests in management interventions to minimize social conflict (Jones et al. 2016). Our findings also supported a process for positioning specific values as a foundation for explaining worries and cares people hold for natural areas, and that acknowledging such values can energize environmental concern (Brehm et al. 2013). Additionally, we encourage and lay groundwork for research that more rigorously evaluates psychometric properties of a scale being used to measure specific values (Carr et al. 2022) and spatial assessment of collective values (Raymond et al. 2014). Despite the difficulty and time-intensity of identifying specific values that drive behavioral decisions, our research approach can inform strategies that resonate with local communities and highlight differences that may exist in the value positions among community members, researchers, federal and state agencies, and others that have vested interests in the outcomes from protected area management.

### Study limitations

There are limitations to our research that require careful consideration when interpreting our findings. First, we combined Alaska Native subsistence users with non-Native subsistence users and instead relied on one question that asked respondents to self-identify as a subsistence user so we could define our survey subgroups. We acknowledge that self-identification does not reflect the history of ways that a respondent experienced Alaskan landscapes and/or interacted with other community or tribal members. Moreover, while we acknowledge different historical and cultural contexts of subsistence use for Alaska Natives and non-Native settlers (Twitchell 2005; Berkes 2009), we lacked sufficient participation of Alaska Natives for a statistically valid comparison. Secondly, our approach to measuring EUH may have increased the error variance related to our analyses. We relied on one variable given its longstanding history in research involving individual experiences within the boundaries of parks and protected areas. We also understand that using one variable to reflect previous experience may not give justice to Indigenous communities that rely on more collective thinking about protected areas that blend their experiences with landscapes in an historical context (Norris 2006). Finally, our findings may also be limited by our conceptualization of PEB, insofar as this construct is heavily influenced by Euro-American contexts which might not translate cleanly to rural Alaska (Aziz and Anjum 2025).

Despite responding to calls in previous research to co-create knowledge with local communities, generate implications to guide environmental agencies’ efforts, and develop sustainable solutions for problems rooted in human behavior (Knapp et al. 2014; Gross et al. 2023; Pascual et al. 2023), our study did not empirically consider factors influencing who can speak on behalf of a community, a matter which has been and remains important for conducting research with Indigenous people. In this vein, we could have gone further to represent the array of voices in the Denali region, particularly Alaska Natives. The two predominantly Native communities of Nikolai and Telida were not included in our survey research process because they were only accessible by plane. Our research team attempted to visit during fieldwork but were prevented from doing so due to heavy wildfire smoke and limitations on funding available to attempt another flight to visit this community. We did engage select individuals from Nikolai during earlier qualitative phases of our project (Salcido et al. 2023). however, the findings in the present study only reflected the perspectives of residents living in Cantwell.

We speculate that, had we engaged individuals from these off-road communities, we may have found relationships that would broaden the concepts of study within the context of subsistence use (Davenport et al. 2024). Indigenous peoples generally would not be inclined to distinguish behavior in a national park vs behavior in other types of lands (Smith and Wishnie 2000), thus our study’s assessment of pro-environmental behavior and its sub-dimensions may appear as park-centered claims (Sahlins 2022) in need of spiritual and/or cultural contexts (Gartler 2018). As a future remedy for inclusion of off-road communities, study design—from research questions to budget implications—should be purposeful about inclusion of Indigenous communities. Such a strategy would entail focused effort to engage Indigenous communities within a representation justice framework, possibly influencing the research protocol including questions asked and scales applied (Davenport et al. 2024).

## Conclusion

This study seeks to illustrate the complexity of relationships among factors predicting PEB among communities living adjacent to protected areas in Alaska. Nature-based settings provide different things to different people, so accounting for the plurality of values invested in a landscape by multiple interests is fundamentally important for the successful cultivation of behavior change. Our study specifically showcases the value basis of subsistence use as being distinguishable and important for decision-makers to represent a diverse constituency more meaningfully through environmental management efforts. Our research additionally supports multidimensional conceptualization of PEB with evidence that previous experience and environmental concerns positively influence public and social-sphere behavior. Finally, we seek to improve measurement of specific values and share insights for future research and practice to promote more sustainable environmental use and stewardship.

## Supplementary Information

Below is the link to the electronic supplementary material.

Supplementary file1 (PDF 4623 KB)
