# Supplementary material for: Values of protected area landscapes shape the behaviors of subsistence users in Interior Alaska
Source: Ambio. 2025 Aug 9;55(1):130–46. doi: 10.1007/s13280-025-02224-7 (PMC12672968; doi:10.1007/s13280-025-02224-7)
Supplement: Supplementary file 1 — Supplementary file1 (PDF 4623 KB) [file 13280_2025_2224_MOESM1_ESM.pdf]

Values of protected area landscapes shape the behaviors of subsistence users in Interior Alaska

Evan L. Salcido[1, 2]
Carena J. van Riper[1]*
William P. Stewart[3]
Christopher M. Raymond[4, 5, 6]
Henry S. Pollock[1,7]

[1]University of Illinois at Urbana-Champaign, Department of Natural Resources and Environmental Sciences, 1102 S. Goodwin Avenue Urbana, IL, 61801, USA

[2]University of Maine, School of Forest Resources, 223A Nutting Hall, Orono, ME 04469, USA

[3]University of Illinois at Urbana-Champaign, Department of Recreation, Sport and Tourism, 104 George Huff Hall, 1206 S 4th St, 1206 S 4th St, Champaign, IL 61820, USA

[4]Helsinki Institute of Sustainability Science (HELSUS), University of Helsinki, PO Box 65, 00014, Finland

[5] Department of Economics and Management, Faculty of Agriculture and Forestry, University of Helsinki, PO Box 65, 00014, Finland

[6]Ecosystems and Environment Research Program, Faculty of Biological and Environmental Sciences, University of Helsinki, PO Box 65, 00014, Finland

[7]Southern Plains Land Trust, P.O. Box 1016, Lamar, Colorado 81052, USA

*Corresponding author
Phone: +1 217-244-9317
email: cvanripe@illinois.edu

Category: Research Article
Word Count: 6,991

Appendix A: Survey questionnaire

A Survey of Residents in Alaska:

Understanding your experiences and preferences for public land management

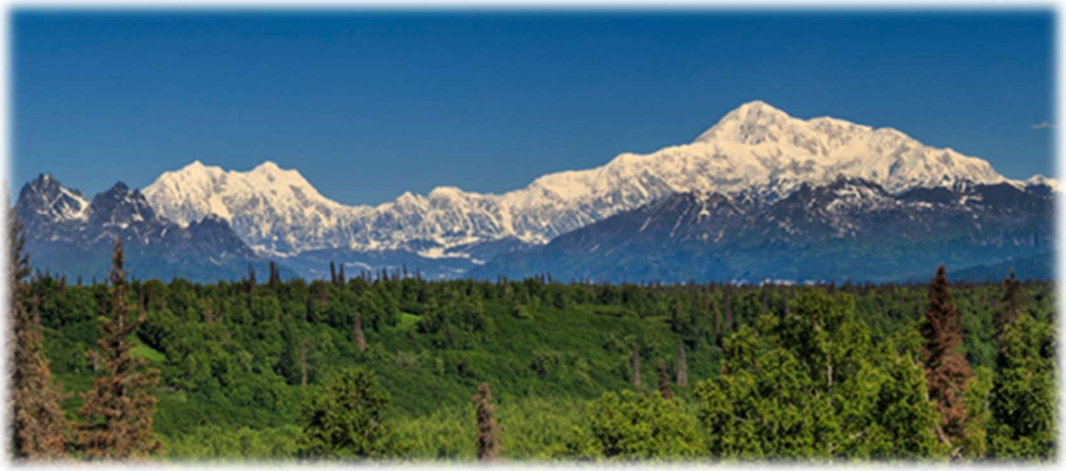

Alaska is a great place to live, yet there are many changes influencing the landscape. To understand how residents like you are responding to these changes, the University of Illinois is partnering with local organizations to learn more about your opinions and experience. You are one of a small number of people chosen for this study, because you live in the region. Your response is important to us. Results from this research will be made publicly available and shared with community leaders and decision-makers. All personal information will be kept confidential and your participation is voluntary.

Please answer each question carefully and save any additional comments for the final page. This questionnaire will take about 20 minutes to complete.

ENVISION

https://publish.illinois.edu/inclusive-conservation-in-denali/

Section 1 of 5: Your Background and Views of the Landscape

In this section, we ask you to provide information about yourself and your views of public land management.

1. **How many years have you been living in Alaska?** _____Years

2. **How many times in your life have you visited Denali National Park?** _____Times

3. **How many times in your life have you visited public lands in Alaska?** _____Times

4. **How many times have you visited public lands in Alaska in the last 12 months?** _____Times

5. **Where have you learned about issues related to public land management in the area where you live?** (✓ all that apply)

- ❒ Public agencies
- ❒ Government officials
- ❒ Scholarly articles
- ❒ Professional societies
- ❒ Environmental groups
- ❒ Government websites
- ❒ Online newspapers
- ❒ Hunting/trapping organizations
- ❒ Social media
- ❒ Webinars
- ❒ Public meetings
- ❒ Friends and family

6. **How frequently have you engaged in the following activities over the past 12 months?**	Very Rarely	Rarely	Occasionally	Frequently	Very Frequently
a. Took measures like re-purposing products to reduce my waste	❒	❒	❒	❒	❒
b. Avoided traveling out of town for non-local products	❒	❒	❒	❒	❒
c. Looked up scientific information about the environment	❒	❒	❒	❒	❒
d. Participated in a policy process like a public comment period that affected the environment	❒	❒	❒	❒	❒
e. Donated money with the intention of benefiting the environment	❒	❒	❒	❒	❒
f. Wrote a letter or email about an environmental issue	❒	❒	❒	❒	❒
g. Encouraged other people to attend an event related to the environment	❒	❒	❒	❒	❒
h. Talked to other people about the environment	❒	❒	❒	❒	❒
i. Learned from other people like longtime residents or Elders to solve an environmental problem	❒	❒	❒	❒	❒

7. We would like to understand the extent to which resident's perspectives are reflected in decisions being made about Denali National Park and Preserve. How could the process for including your opinions in decision-making be improved?

____

____

____

____

____

8. We would like to understand why you think the landscape around your home is different from other places. Please use the space below to describe why, if at all, the place where you live is special.

____

____

____

____

____

**9. There are many ways a place could be considered distinctive. Below we describe some of the ways you might think about the place where you live. To what extent do you agree with <u>each</u> of the following statements about why this place is special?**	Strongly Disagree	Disagree	Neutral	Agree	Strongly Agree
a. **Economic:** A place to earn income for employment	❐	❐	❐	❐	❐
b. **Subsistence:** A place to harvest food or other resources to sustain my life and that of my family	❐	❐	❐	❐	❐
c. **Education:** A place to learn about, teach, or research the environment and people	❐	❐	❐	❐	❐
d. **Recreation:** A place where I can pursue recreation activities	❐	❐	❐	❐	❐
e. **Family:** A place where I can spend time with my family	❐	❐	❐	❐	❐
f. **Rejuvenation:** A place where I can feel better physically and/or mentally	❐	❐	❐	❐	❐
g. **Sense of community:** A place where I have close relationships with other members of my community	❐	❐	❐	❐	❐
h. **Heritage:** A place with history and traditions that are passed down to future generations	❐	❐	❐	❐	❐
i. **Spirituality:** A place that is sacred, religious, or spiritually significant	❐	❐	❐	❐	❐
j. **Aesthetics:** A place that has attractive scenery, sights, sounds, or smells that cannot be experienced anywhere else	❐	❐	❐	❐	❐
k. **Ecological Integrity:** A place that has intact ecosystems with the ability to support and maintain ecological processes	❐	❐	❐	❐	❐
l. **Wildlife:** A place inhabited by wildlife unique to Alaska	❐	❐	❐	❐	❐

Section 2 of 5: Future Management Scenarios

Landscapes in Interior Alaska are rapidly changing in ways that impact local residents. In this section, we ask you questions about possible changes to landscape conditions that may occur **over the next 30 years** in the area where you live. For each question, please indicate your preferences for the future by choosing between two possible future scenarios or the current condition. Each scenario includes three or five environmental “features” that represent conditions influenced by different management agencies and potential policies. These features are described below.

Please read this material carefully.

**Moose Population**<br>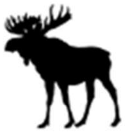	The total number of moose in the largest game management unit of Interior Alaska is estimated to be 55,000. These numbers are expected to increase due to fires that create open spaces, providing ideal moose habitat.<br><br>This feature is set at three levels that may occur **over the next 30 years:**<br>1. **0% increase in moose population**<br>2. **15% increase in moose population**<br>3. **30% increase in moose population**
**Off-season Tourism**<br>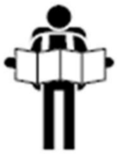	Off-season tourism is the number of out of town visitors who travel to Interior Alaska from October – April. Over the past 10 years, visitation has varied, but increased by about 20%. During off-season months in 2019, there were 19,332 visitors to Denali National Park and Preserve.<br><br>This feature is set at three levels that may occur **over the next 30 years:**<br>1. **25% decrease in off-season tourism growth rate**<br>2. **Maintain current off-season tourism growth rate**<br>3. **25% increase in off-season tourism growth rate**
**Acres Managed for Fire**<br>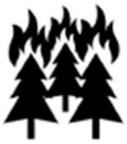	Fire protection is provided by several public land management agencies. Currently, about 10% (10,581,937 acres) of all forested acres in Interior Alaska are managed to a limited degree.<br><br>This feature is set at three levels of acres that could be managed **over the next 30 years:**<br>1. **0% increase in acres managed**<br>2. **15% increase in acres managed**<br>3. **30% increase in acres managed**
**Annual Cost**<br>**$**	Residents of Alaska receive payments each year from the Alaska Permanent Fund. In 2019, residents accepted into this program each received $1,606. To minimize impacts from the other features described above, everyone’s dividend could be reduced. We would like to know how much you would be willing to pay from this fund to prevent changes to the landscape in Interior Alaska. This feature is set at five levels ranging from **$0 to $100** **over the next 30 years.**

Each scenario below is independent and includes three options. Please select the option that you would prefer for the area where you live.

Future Scenario 1

Suppose Option A and Option B were the *only* options available besides "No change" over **the next 30 years**. Which would you choose? *Please check the box that represents your choice.*

Attribute	Moose population	Off-season Tourism	Acres Managed for Fire	Annual Cost $	I would choose
**Option A**	**15% Increase**	**25% Decrease in growth rate**	**15% Increase**	**$0**	❒ A
**Option B**	**15% Increase**	**25% Increase in growth rate**	**15% Increase**	**$75**	❒ B
**Option C**	**No change**				❒ C

Future Scenario 2

Suppose Option A and Option B were the *only* options available besides "No change" over **the next 30 years**. Which would you choose? *Please check the box that represents your choice.*

Attribute	Moose population	Off-season Tourism	Acres Managed for Fire	Annual Cost $	I would choose
**Option A**	**0% Increase**	**25% Increase in growth rate**	**15% Increase**	**$100**	❒ A
**Option B**	**30% Increase**	**25% Decrease in growth rate**	**15% Increase**	**$0**	❒ B
**Option C**	**No change**				❒ C

Future Scenario 3

Suppose Option A and Option B were the *only* options available besides "No change" over **the next 30 years**. Which would you choose? *Please check the box that represents your choice.*

Attribute	Moose population	Off-season Tourism	Acres Managed for Fire	Annual Cost $	I would choose
**Option A**	**30% Increase**	**25% Decrease in growth rate**	**30% Increase**	**$25**	❒ A
**Option B**	**0% Increase**	**25% Increase in growth rate**	**0% Increase**	**$75**	❒ B
**Option C**	**No change**				❒ C

Future Scenario 4

Suppose Option A and Option B were the *only* options available besides "No change" over **the next 30 years**. Which would you choose? *Please check the box that represents your choice.*

Attribute	Moose population	Off-season Tourism	Acres Managed for Fire	Annual Cost $	I would choose
**Option A**	**15% Increase**	**25% Increase in growth rate**	**0% Increase**	**$50**	❒ A
**Option B**	**15% Increase**	**25% Decrease in growth rate**	**30% Increase**	**$50**	❒ B
**Option C**	**No change**				❒ C

Future Scenario 5

Suppose Option A and Option B were the *only* options available besides "No change" over **the next 30 years**. Which would you choose? *Please check the box that represents your choice.*

Attribute	Moose population	Off-season Tourism	Acres Managed for Fire	Annual Cost	I would choose
**Option A**	**30% Increase**	**Maintain current growth rate**	**0% Increase**	**$75**	❒ A
**Option B**	**0% Increase**	**Maintain current growth rate**	**30% Increase**	**$0**	❒ B
**Option C**	**No change**				❒ C

Future Scenario 6

Suppose Option A and Option B were the *only* options available besides "No change" over **the next 30 years**. Which would you choose? *Please check the box that represents your choice.*

Attribute	Moose population	Off-season Tourism	Acres Managed for Fire	Annual Cost	I would choose
**Option A**	**0% Increase**	**Maintain current growth rate**	**15% Increase**	**$0**	❒ A
**Option B**	**30% Increase**	**Maintain current growth rate**	**15% Increase**	**$100**	❒ B
**Option C**	**No change**				❒ C

Section 3 of 5: Your Attitudes and Beliefs

This section includes questions about the features from the future management scenarios in the previous section, including moose population, off-season tourism, fire management, and annual costs from the Alaska Permanent Fund. We also ask about your beliefs related to global warming.

**10. We would like to understand your attitudes toward key features of landscape change. To what extent do you agree with the following statements for the area where you live?**	Strongly Disagree	Disagree	Neutral	Agree	Strongly Agree
a. I like knowing that there are healthy populations of moose	❐	❐	❐	❐	❐
b. It is important that others in my community see moose	❐	❐	❐	❐	❐
c. The opportunity to hunt moose is an important part of living in my community	❐	❐	❐	❐	❐
d. Fire protection provided by public land management agencies reduces the chance of high-intensity wildfires	❐	❐	❐	❐	❐
e. Increasing numbers of forest fires pose as a serious threat to my way of life	❐	❐	❐	❐	❐
f. The number of forested acres managed for fire protection should be increased	❐	❐	❐	❐	❐
g. Off-season tourism (October-April) supports economic well-being	❐	❐	❐	❐	❐
h. Increases in off-season tourism are extremely beneficial for my community	❐	❐	❐	❐	❐
i. My personal quality of life would increase with more off-season tourism	❐	❐	❐	❐	❐
j. Dividends from the Alaska Permanent Fund benefit all generations of Alaskans	❐	❐	❐	❐	❐
k. Reductions in the amount of money per dividend would negatively impact local communities	❐	❐	❐	❐	❐
l. I support the reduction of my dividend from the Alaska Permanent Fund to benefit the environment	❐	❐	❐	❐	❐

11. Global warming refers to the idea that the world's average temperature has been increasing over the past 150 years and may increase more in the future. Do you think that global warming is happening?

❐ Yes ❐ No ❐ Don't know

→ **11b. If yes, how sure are you that global warming is happening?**

❐ Not at all sure ❐ Somewhat sure ❐ Moderately sure ❐ Very sure ❐ Extremely sure

11c. Do you think global warming is…?

❐ Caused mostly by human activities ❐ Caused mostly by natural changes in the environment ❐ None of these because global warming is not happening

Section 4 of 5: Your Values and Environmental Concerns

We would like to better understand your personal values and concerns about the environment. This information is helpful to understand the things you care about most. Please only provide the information that you are comfortable sharing.

12. These questions are about your personal values. Please rate the extent to which you consider each value to be a guiding principle in your life.

	Unimportant	Of Little Importance	Moderately Important	Important	Very Important
a. **Protecting the environment**: preserving nature	❐	❐	❐	❐	❐
b. **Unity with nature**: fitting into nature	❐	❐	❐	❐	❐
c. **A world of beauty**: beauty of nature and the arts	❐	❐	❐	❐	❐
d. **Equality**: equal opportunity for all	❐	❐	❐	❐	❐
e. **Social justice**: correcting injustice, care for others	❐	❐	❐	❐	❐
f. **A world at peace**: free of war and conflict	❐	❐	❐	❐	❐
g. **Authority**: the right to lead or command	❐	❐	❐	❐	❐
h. **Social power**: control over others, dominance	❐	❐	❐	❐	❐
i. **Influential**: having an impact on people and events	❐	❐	❐	❐	❐
j. **Fulfilment of desire**: food, fun, pleasure	❐	❐	❐	❐	❐
k. **Enjoying life**: pursuing hobbies, leisure, socializing	❐	❐	❐	❐	❐
l. **Reducing worries**: seeking comfort and relaxation	❐	❐	❐	❐	❐
m. **Personal growth**: development of new skills, learning, or gaining insight into something	❐	❐	❐	❐	❐
n. **Pursuit of excellence**: attaining a personal ideal in life	❐	❐	❐	❐	❐
o. **Autonomy**: deciding your own future and doing what you believe in	❐	❐	❐	❐	❐
p. **Satisfaction with life**: finding meaning, value, and relevance to a broader context	❐	❐	❐	❐	❐

13. I am morally obligated to minimize environmental impacts on public lands near my home.

❐	❐	❐	❐	❐
Strongly Disagree	Disagree	Neutral	Agree	Strongly Agree

14. I would feel guilty if I negatively impacted public lands near my home.

❐	❐	❐	❐	❐
Strongly Disagree	Disagree	Neutral	Agree	Strongly Agree

15. People like me should be proud if they can limit their impact on public lands near my home.

❐	❐	❐	❐	❐
Strongly Disagree	Disagree	Neutral	Agree	Strongly Agree

**16. There are many different perspectives on how society should be organized. How strongly do you agree with the following statements?**	Strongly Disagree	Disagree	Neutral	Agree	Strongly Agree
a. When I have problems, I try to solve them on my own	❐	❐	❐	❐	❐
b. I prefer tasks where I work something out on my own	❐	❐	❐	❐	❐
c. The freedom of an individual should not be limited	❐	❐	❐	❐	❐
d. There are limitations in life that we have to accept whether we want to or not	❐	❐	❐	❐	❐
e. There is no use in doing things for other people – you only get taken advantage of	❐	❐	❐	❐	❐
f. I would not participate in civic action groups. Those in power do what they want anyway	❐	❐	❐	❐	❐
g. It is important to preserve our customs and traditions	❐	❐	❐	❐	❐
h. I prefer clear instruction from my supervisors about what to do	❐	❐	❐	❐	❐
i. In a family, adults and children should have different degrees of influence on decisions	❐	❐	❐	❐	❐
j. Firms and institutions should be organized in a way that everybody can influence important decisions	❐	❐	❐	❐	❐
k. In the case of important issues for a family, everyone should contribute to decisions	❐	❐	❐	❐	❐
l. Important questions for our society should not be decided upon by experts, but by the people	❐	❐	❐	❐	❐

**17. We would like to understand more about your concern for the environment. How strongly do you agree with the following statements?**	Strongly Disagree	Disagree	Neutral	Agree	Strongly Agree
a. It bothers me when I think about the environmental conditions in which our children and grandchildren will probably have to live in	❐	❐	❐	❐	❐
b. If we continue down the same path, we are heading toward an environmental catastrophe	❐	❐	❐	❐	❐
c. Decision-makers are doing far too little to protect the environment	❐	❐	❐	❐	❐
d. To protect the environment, we should all be willing to reduce our current standard of living	❐	❐	❐	❐	❐
e. In my opinion, many environmental threats are exaggerated	❐	❐	❐	❐	❐
f. There are limits on growth that our industrialized world has already exceeded or will soon reach	❐	❐	❐	❐	❐

18. When you think about management of Denali National Park and Preserve, which organizations have the most influence on decisions and why?

____

____

____

Section 5 of 5: About You

Our final questions are about your socio-demographic characteristics. Please enter only the information that you are comfortable sharing.

19. What is your gender? ❒ Female ❒ Male

20. What is your age? ______________________________

21. What is the name of the community where you live? ______________________

22. "Subsistence" is when residents use wild, renewable resources (such as hunting or gathering) for personal consumption.

22a. Do you identify as a subsistence user? ❒ Yes ❒ No

22b. How important is subsistence use to you?

❒	❒	❒	❒	❒
Not at all Important	Slightly Important	Moderately Important	Very Important	Extremely Important

23. With which racial group(s) do you identify? (Please ✓ all that apply)

- ❒ American Indian and Alaska Native
- ❒ Asian
- ❒ White
- ❒ Black or African American
- ❒ Pacific Islander
- ❒ Other: ____________

24. What is your annual household income before taxes? (Please ✓ one)

- ❒ Less than $24,999
- ❒ $25,000-$49,999
- ❒ $50,000-$99,999
- ❒ $100,000-$149,999
- ❒ $150,000-$199,999
- ❒ $200,000-$249,999
- ❒ $250,000 or more
- ❒ Prefer not to answer

25. What is the highest level of education you have completed? (Please ✓ one)

- ❒ Some high school
- ❒ High school graduate or GED
- ❒ Two-year degree
- ❒ Bachelor's degree
- ❒ Professional certificate
- ❒ Graduate degree

Are you interested in continuing to participate in this research?

We are designing an online program to learn more about how protected areas and communities are changing in Alaska. We are looking for people to enroll in this program! **Monetary incentives will be provided.** If you are interested in sharing thoughts about the environment while exchanging ideas with others in your community, please provide your information below.

Name: ______________________________

Email address and/or phone number: ______________________________

Thanks for your participation!

Use the space below to share any additional thoughts about this study and indicate if you would like a copy of our final report.

Please place the completed questionnaire into the addressed postage-paid return envelope and mail it back to us.

If you have any questions, please contact:

Carena van Riper
University of Illinois at Urbana-Champaign
Department of Natural Resources and Environmental Sciences
Email: cvanripe@illinois.edu
Project website: https://publish.illinois.edu/inclusive-conservation-in-denali/

Institutional Review Board Approval: 18679
Expiration Date: May 25th, 2023

Appendix B: Missing data statistics for pooled social value, previous experience, environmental concern, and pro-environmental behavior data

Component	Complete Cases	Missing Cases		Estimated Statistics	
		Count	*Percent*	*M*	*SD*
Pristine Nature[1a]	315	17	5.1	4.50	0.62
Aesthetics	318	14	4.2	4.53	0.76
Ecological Integrity	316	16	4.8	4.36	0.80
Wildlife	318	14	4.2	4.60	0.72
Communal Cohesion[1a]	305	27	8.1	3.79	0.71
Heritage	315	17	5.1	3.63	1.01
Spirituality	317	15	4.5	3.48	1.13
Sense of Community	317	15	4.5	3.96	1.02
Subsistence	313	19	5.7	4.08	0.86
Relational Fulfillment[1a]	315	17	5.1	4.45	0.57
Family	317	15	4.5	4.25	0.89
Recreation	318	14	4.2	4.58	0.59
Rejuvenation	316	16	4.8	4.52	0.76
Capacity Growth[1a]	310	22	6.6	3.65	0.78
Economic	314	18	5.4	3.34	1.10
Education	315	17	5.1	3.97	0.88
Previous Experience[2a]	302	30	9.0	2.38	1.07
Environmental Concern[1b] ($\alpha = 0.91$)	300	32	9.6	3.88	0.98
1. It bothers me when I think about the environmental conditions in which our children and grandchildren will probably have to live in	310	22	6.6	4.08	0.99
2. If we continue down the same path, we are heading toward an environmental catastrophe	312	20	6.0	3.93	1.18
3. Decision-makers are doing far too little to protect the environment	313	19	5.7	3.98	1.18
4. To protect the environment, we should all be willing to reduce our current standard of living	309	23	6.9	3.48	1.32
5. There are limits on growth that our industrialized world has already exceeded or will soon reach	309	23	6.9	3.86	0.99

[1]Measured along a Likert scale where 1 = "Strongly Disagree" and 5 = "Strongly Agree."
[2]Measured along a scale where 1 = "Low"; 2 = "Moderate"; 3 = "High"; and 4 = "Very High"
a. Little's MCAR test: Chi-Square = 189.86, df = 181, $p > 0.05$
b. Little's MCAR test: Chi-Square = 34.69, df = 25, $p > 0.05$

Appendix B (continued): Missing data statistics for pooled social value, previous experience, environmental concern, and pro-environmental behavior data

Component	Complete Cases	Missing Cases		Estimated Statistics	
		Count	*Percent*	*M*	*SD*
Pro-Environmental Behavior: Private[3c] (α = 0.56)	313	19	5.7	3.66	0.78
1. Took measures like re-purposing products to reduce my waste	322	10	3.0	4.09	0.98
2. Avoided traveling out of town for non-local products	314	18	5.4	3.48	1.13
3. Looked up scientific information about the environment	321	11	3.3	3.39	1.13
Pro-Environmental Behavior: Public[3c] (α = 0.80)	319	13	3.9	2.39	1.03
4. Participated in a policy process like a public comment period that affected the environment	320	12	3.6	2.51	1.20
5. Donated money with the intention of benefiting the environment	321	11	3.3	2.47	1.23
6. Wrote a letter or email about an environmental issue	321	11	3.3	2.19	1.23
Pro-Environmental Behavior: Social[3c] (α = 0.74)	314	18	5.4	2.89	0.90
7. Encouraged other people to attend an event related to the environment	321	11	3.3	2.22	1.12
8. Talked to other people about the environment	318	14	4.2	3.72	1.08
9. Learned from other people like longtime residents or Elders to solve an environmental problem	319	13	3.9	2.72	1.13

[3]Measured along a Likert scale where 1 = "Very Rarely" and 5 = "Very Frequently."
c. Little's MCAR test: Chi-Square = 33.88, df = 73, $p > 0.05$

Appendix C: Tests of normality, skewness, and kurtosis for pooled social value, previous experience, environmental concern, and pro-environmental behavior data

Variable	Shapiro–Wilk	Skewness	Kurtosis
Pristine Nature	0.77*	-1.70	3.86
Aesthetics	0.62*	-1.94	4.42
Ecological Integrity	0.74*	-1.18	0.89
Wildlife	0.61*	-2.20	5.70
Communal Cohesion	0.96*	-0.62	0.81
Heritage	0.89*	-0.48	-0.14
Spirituality	0.90*	-0.36	-0.45
Sense of Community	0.83*	-1.10	1.10
Subsistence	0.81*	-0.98	1.03
Relational Fulfillment	0.85*	-1.13	1.69
Family	0.79*	-1.16	1.07
Recreation	0.64*	-1.37	2.23
Rejuvenation	0.64*	-2.15	6.20
Capacity Growth	0.94*	-0.63	0.44
Economic	0.89*	-0.48	-0.44
Education	0.84*	-0.74	0.53
Previous Experience	0.87*	0.13	-1.24
Environmental Concern	-	-	-
It bothers me when I think about the environmental conditions in which our children and grandchildren will probably have to live	0.82*	-1.02	0.54
If we continue down the same path, we are heading toward an environmental catastrophe	0.81*	-0.98	0.00
Decision-makers are doing far too little to protect the environment	0.79*	-1.02	0.04
To protect the environment, we should all be willing to reduce our current standard of living	0.86*	-0.53	-0.90
There are limits on growth that our industrialized world has already exceeded or will soon reach	0.86*	-0.74	-0.03
Pro-Environmental Behavior: Public	-	-	-
Participated in a policy process like a public comment period that affected the environment	0.89*	0.32	-0.75
Donated money with the intention of benefiting the environment	0.89*	0.45	-0.68
Wrote a letter or email about an environmental issue	0.84*	0.67	-0.63
Pro-Environmental Behavior: Social	-	-	-
Encouraged other people to attend an event related to the environment	0.86*	0.47	-0.75
Talked to other people about the environment	0.87*	-0.74	0.21
Learned from other people like longtime residents or Elders to solve an environmental problem	0.90*	0.06	-0.60

*$p < 0.05$; indicates a non-normal distribution

Appendix D: Correlation coefficient matrix of twelve specific values items

	1	2	3	4	5	6	7	8	9	10	11	12
1. Economic	1.00											
2. Subsistence	0.06	1.00										
3. Education	0.21	0.10	1.00									
4. Recreation	0.09	0.27	0.29	1.00								
5. Family	0.11	0.20	0.15	0.28	1.00							
6. Rejuvenation	0.03	0.24	0.36	0.42	0.31	1.00						
7. Sense of community	0.19	0.18	0.24	0.26	0.22	0.43	1.00					
8. Heritage	0.14	0.33	0.25	0.16	0.16	0.23	0.42	1.00				
9. Spirituality	0.04	0.26	0.31	0.13	0.15	0.38	0.29	0.52	1.00			
10. Aesthetics	-0.01	0.22	0.29	0.38	0.13	0.42	0.20	0.27	0.32	1.00		
11. Ecological integrity	-0.05	0.19	0.42	0.27	0.07	0.33	0.14	0.24	0.28	0.49	1.00	
12. Wildlife	-0.05	0.18	0.28	0.22	0.14	0.29	0.13	0.23	0.28	0.52	0.56	1.00

Appendix E: Loadings, eigenvalues, and variation explained based on a principal component analysis with varimax rotation for 12 specific values items. Bolded items indicate the variables associated with each component.

Variable	Component			
	Pristine Nature	*Communal Cohesion*	*Relational Fulfillment*	*Capacity Growth*
Economic	-0.13	0.07	0.09	**0.83**
Subsistence	0.05	**0.49**	0.40	-0.26
Education	0.50	0.13	0.18	**0.57**
Recreation	0.34	-0.07	**0.73**	0.01
Family	-0.05	0.12	**0.71**	0.11
Rejuvenation	0.37	0.25	**0.62**	0.11
Sense of Community	0.03	**0.49**	0.45	0.25
Heritage	0.15	**0.84**	0.06	0.11
Spirituality	0.30	**0.75**	0.02	0.07
Aesthetics	**0.74**	0.16	0.25	-0.05
Ecological Integrity	**0.82**	0.13	0.05	0.07
Wildlife	**0.78**	0.16	0.08	-0.08
Eigenvalue	2.46	1.92	1.88	1.19
Variation explained (%)	20.51	15.99	15.70	9.89

Note. Bolded values indicate the variables associated with each component.

Appendix F: Invariance constraints and chi-square difference testing between subsistence users and non-subsistence users

	χ^2	df	$\Delta\chi^2$	Δdf	P - value
1. Configural fit	338.88	182	-	-	-
2. Constrained factor loadings	343.94	190	5.06	8	0.74
3. Constrained intercepts	349.48	198	5.54	8	0.71
4. Constrained residuals	362.25	209	12.77	11	0.64
